# Full-Length Transcriptome Sequences Provide Insight Into Hermaphroditism of Freshwater Pearl Mussel *Hyriopsis schlegelii*

**DOI:** 10.3389/fgene.2022.868742

**Published:** 2022-03-24

**Authors:** Qi Zeng, Beijuan Hu, Andres Hortas Blanco, Wanchang Zhang, Daxian Zhao, Paulino Martínez, Yijiang Hong

**Affiliations:** ^1^ School of Life Sciences, Nanchang University, Nanchang, China; ^2^ Key Lab of Aquatic Resources and Utilization of Jiangxi, Nanchang, China; ^3^ Key Laboratory of Poyang Lake Environment and Resource Utilization, Ministry of Education, Nanchang, China; ^4^ Department of Zoology Genetics and Physical Anthropology, Faculty of Veterinary, University of Santiago de Compostela, Lugo, Spain

**Keywords:** Hyriopsis schlegelii, Iso-seq, RNA-seq, gonad, *17β-hsd2*

## Abstract

The freshwater mussel *Hyriopsis schlegelii* is a cultured bivalve in China, and the quality of the pearls produced is affected by the type of gonads. However, because of the lack of a published genome and the complexity of sex determination, research on sex reversal and development of this species is limited. In this study, Illumina RNA-seq and PacBio Isoform Sequencing (Iso-Seq) were combined to analyze the gonads of *H. schlegelii*. A total of 201,481 high-quality transcripts were generated. The study identified 7,922 differentially expressed genes in three comparison group (females versus males, hermaphrodites versus females, and hermaphrodites versus males). Twenty-four genes were identified as potential sex-related genes, including *sox9* and *wnt4* involved in sex determination, and *vtg*, *cyp17a1* and *17β-hsd2* involved in gonadal development. We also speculated a possible pathways for the formation of hermaphroditism in *H. schlegelii*. The data provide a clear view of the transcriptome for *H. schlegelii* gonads and will be valuable in elucidating the mechanisms of gonad development.

## Introduction

The *Hyriopsis schlegelii* is the main freshwater cultured pearl mussel in China. It is a freshwater pearl-producing bivalve originally endemic to Lake Biwa in central Japan. This species was known worldwide as the Biwa pearl mussel but is now threatened in its original habitat and classified as a critically endangered species in the Japanese Red Data Book (Ministry of the Environment 2005).

Many bivalves are economically important species specifically bred to produce pearls. Pearl oysters include *Pinctada margaritifera*, *Pinctada maxima*, *Pinctada fucata*, and *Pteria penguin* (Adzigbli et al., 2019); freshwater pearl mussels include *Hyriopsis cumingii* (Zhao et al., 2013) and *Hyriopsis schlegelii* (Wang et al., 2016). Pearls are produced through an implantation operation in which the nucleus with mantle tissue from the donor oyster or mussel is inserted into the gonad of the host oyster or mussel. The sex of the mussel has an impact on the quality of the pearl. Compared with the female, the male produces higher value pearls. Male pearls have uniform thickness and smoothness, whereas female pearls often have multiple scratches (Adzigbli et al., 2019). Furthermore, if female freshwater pearl mussels are cultured separately, the quality of female pearls can be improved (Zhao et al., 2013). Therefore, it is significant to determine the mechanism of sex regulation for reproductive evaluation and aquaculture management.

Sex steroid hormones play vital roles in sexual differentiation, maintenance of sexual characteristics, gametogenesis and gamete maturation. In bivalves, sex steroid hormones are related to reproduction, and they promote the growth and development of gametes by affecting gametogenesisand related pathways (Croll and Wang, 2007). In *Ruditapes decussatus*, progesterone, testosterone, and estradiol are detected, and the content of these three hormones changes with the different stages of gametes (Ketata et al., 2007). Similarly, the content of progesterone changes with the reproductive cycle in *Mya arenaria* (Siah et al., 2002). During the reproductive process of *Chlamys farreri*, testosterone in males and 17β-estradiol in females is similar to the changing trend of the sex gland cell index that has an endocrine regulatory function (Zheng et al., 2014). It is noteworthy that an injection of estradiol, testosterone, progesterone, or dehydroepiandrosterone (DHEA) accelerated gonadal differentiation of *Placopecten magellanicus*. Estradiol stimulates the growth of oocytes, and testosterone degrades the development of female gonads (Wang and Croll, 2004). By following the dynamic changes of progesterone, testosterone, and estradiol in the scallop *Argopecten irradians,* significant differences were observed between testosterone and estradiol in the ovary and testis (Zhang et al., 2021). It has been speculated that these two hormones may be involved in the sex differentiation of scallops.

Hermaphroditism occurs during gonadal development of *H. schlegelii* as in other bivalves, such as *Arconaia lanceolata* (Wu et al., 2018), *Pinctada Margaritifera* (Teaniniuraitemoana et al., 2014), *Tridacna squamosa* (Li et al., 2020), and *Crassostrea gigas* (Broquard et al., 2021). Hermaphrodite bivalves act as egg-producing females or sperm-producing males at least once in their life cycle. The double-sex pattern can be implemented at the same time or at different times. In *H. schlegelii*, hermaphroditism occurs in the development stage of 26–32 months. Male and female follicular tissues coexist in hermaphroditic individuals, and male follicular tissues account for more than 90% of the gonadal tissues (Wu et al., 2017). However, the mechanism of this phenomenon is currently unknown.

In the present study, the full-length transcriptome of *H. schlegelii* was generated using PacBio Isoform Sequencing (Iso-Seq) and RNA-seq analysis. Differentially expressed genes (DEGs) in three different gonadal development conditions were examined by a series of transcriptome RNA-seq of the gonads. To the best of our knowledge, this is the first report of a full-length transcriptome for the gonads in a bivalve. These data provide a clear view of the transcriptome dynamics for *H. schlegelii* gonads and facilitate research on the mechanism of bivalve gonadal development.

## Material and Methods

### Samples Collection and Histological Analysis

In November, a total of 50 30-month-old *H. schlegelii* individuals were collected from Fuzhou Hongmen Reservoir of Jiangxi Province, China. The mussel gonads were immediately dissected. For each mussel, gonad tissues were sampled for RNA extraction (snap frozen in liquid nitrogen) and fixed in neutral poly-methanol stationary solution for histology. All tissues were dehydrated in an ethanol series and embedded in paraffin wax. The 5 μm sections of gonad tissue were stained with hematoxylin and eosin (H&E) according to standard protocols. Sex was identified by the presence of sperms or egg cells in the gonads. If sperms and egg cells both exist in the gonads, it is considered hermaphrodite.

### Sample Preparation and RNA Preparation

The gonads of nine individuals (three males, three females, and three hermaphrodites) were used for short-read Illumina sequencing, and the gonads of two individuals (one female and one male) were used for long-read PacBio sequencing. According to the manufacturer’s instructions, total RNA was extracted using 3 ml of Trizol per sample (Invitrogen, Carlsbad, CA, United States). After treatment with RNase-free DNase I (Promega Madison, WI, United States), three male testes, three female ovaries, and three gonads from a double-sex individual were used for Illunima RNA-seq. Additionally, two samples, a male and a female, were adopted for PacBio Iso-Seq. High-throughput sequencing was performed at the Novogene Bioinformatics Institute (Novogene, Beijing, China).

### Pac-Bio Sequencing

The Iso-Seq method was used for sequencing long-reads of even proportions of tissue from gonads. The quality of extracted RNAs was evaluated using an Agilent 2100 Bioanalyzer (SA Pathology, Adelaide, SA, Australia). High-quality RNAs (RNA integrity number >6.0) were used for cDNA synthesis. The Iso-Seq library was prepared according to the Iso-Seq protocol using Oligo (dT) enriched mRNA. A Clontech SMARTer polymerase chain reaction (PCR) cDNA Synthesis Kit was used to reverse-transcribe mRNA into DNA. The BluePippin system was used to perform fragment screening of the full-length cDNA for damage repair, end repair, the connection of SMRT dumbbell joints, and exonuclease digestion. The Iso-Seq library was prepared as described by Pacific Biosciences (PN 100-092-800-03). Single-Molecule Real-Time (SMRT) sequencing was performed on the PacBio Sequel System.

### Illumina Novaseq Sequencing

Replicate testis and ovary RNA samples were used in mRNA paired-end library construction using a NEBNext^®^ Ultra™ RNA Library Prep Kit for Illumina^®^ (NEB, Ipswich, MA, United States) following the manufacturer’s recommendations. Briefly, mRNA was obtained via poly-A mRNA isolation with oligo-dT beads, and the fragments were used for cDNA synthesis with random hexamer primers (NEB). After end-repair, adenylation, adaptor ligation, cDNA purification, and PCR amplification, 3′ paired-end cDNA libraries were constructed, and their quality was evaluated on the Agilent Bioanalyzer 2100 system. After cluster generation, all libraries were loaded onto an Illumina Novaseq platform, and 150 paired-end reads were generated.

Raw Illumina reads were filtered by removal of low-quality reads and the adapters used for library construction. Reads containing undetermined nucleotides (N) and low-quality reads (Qphred ≥20) accounting for more than 50% of the entire read length were discarded. All downstream analyses were based on the high-quality clean data.

### Error Correction of PacBio Iso-Seq Reads

The Smrtlink (v5.1) program (http://www.pacb.com/support/software-downloads/) was used to process raw data produced by PacBio. We used the subreads.bam file in the offline data to implement the circular consensus sequence (CCS) algorithm (by ccs, parameters: ---minPasses = 2, minPredictedAccuracy = 0.8), to perform self-error correction to obtain CCS sequences (ccs.bam). These were classified by checking the polyA tail and the signals of 5 and 3′ primers to categorize the reads as FLNC (full-length non-chimera) and NFL (non-full-length non-chimera) reads by using pbtranscript classification (parameters: -min_seq_len = 200). We used the isoform-level clustering (ICE) algorithm to cluster FLNC reads and used Arrow to subsequently polish the NFL reads to create the final consensus transcriptome. The consensus Iso-Seq transcriptome was corrected with Illumina short reads using the software LoRDEC (v0.9) (Salmela and Rivals, 2014). Error-corrected consensus reads were clustered by using cd-hit-est from the CD-HIT (v4.6.8) (Fu et al., 2012) program (-c 0.95 -M 0 -T 0 -G 0 -aL 0.90 -AL 100 -aS 0.99 -AS 30) to obtain final transcripts for subsequent analysis.

### Gene Functional Annotation and Structural Analysis

To get comprehensive gene functional annotations of the unique sequences obtained after using the CD-HIT software to eliminate redundancy, we used blastn and blastx to align with NR (NCBI non-redundant protein sequences), NT (NCBI non-redundant nucleotide sequences), Swiss-Prot (a manually annotated and reviewed protein sequence database), and Kyoto Encyclopedia of Genes and Genomes (KEGG) databases, setting the e-value as 1×e^−10^. Transcripts were aligned to the PFAM (Protein family) database to perform annotation using functional domains of proteins via Hmmscan. Finally, annotations were loaded into the Blast2GO (Conesa et al., 2005) program to obtain Gene Ontology (GO) terms. Transcription factors were performed using the animalTFDB 2.0 database by blastx. The Coding-Non-Coding-Index (CNCI) (Sun et al., 2013), Coding Potential Calculator (CPC) (Kong et al., 2007), Pfam-scan (Finn et al., 2016) and PLEK (Li et al., 2014) tools were used to predict the coding potential of transcripts. SSR of the transcriptome were identified using MISA (http://pgrc.ipk-gatersleben.de/misa/misa.html). It allows the identification and localization of perfect microsatellites as well as compound microsatellites which is interrupted by a certain number of bases.

### Quantification of Gene Expression

Clean Illumina data were mapped to the full-length gonad transcriptomes using bowtie2 (Langmead and Salzberg, 2012), and read counts were obtained by RSEM (Li and Dewey, 2011) for each sample. For all comparisons, we considered the effects of sequence depth and gene length on fragments and normalized all read counts to the aligned RPKM (reads per kilobase per million mapped reads) to obtain the relative levels. FPKM >0.3 was defined as the threshold of significant gene expression.

### Identification and Analysis of DEGs

The R package DESeq2 (Love et al., 2014) provides methods to identify DEGs between sex using negative binomial generalized linear models. The resulting *p* values were adjusted using a 5% false discovery rate to correct for multiple tests. Furthermore, a fold change **|**log2 (exp/ref)**|** > 1 was used to consider a gene as differentially expressed. Sets of DEGs and functions were obtained for the following comparisons: females versus males, hermaphrodites versus females, and hermaphrodites versus males.

To explore the potential functions of these DEGs in the three comparison groups, we performed GO and KEGG pathway enrichment analyses. DEGs were distributed into different GO terms based on their associations with cellular components, molecular functions, and biological processes. GO enrichment analysis of DEGs was executed using the clusterProfiler (Yu et al., 2012) R package (v3.14.3) based on the hypergeometric distribution. A *Padj* value <0.05 was assumed to indicate enriched functions. KOBAS software (3.0) (Bu et al., 2021) was used to find KEGG pathways in the statistical enrichment of DEGs.

## Result

### Histology Characteristics From the Gonads of 30-month-Old *H. schlegelii*

**FIGURE 1:**
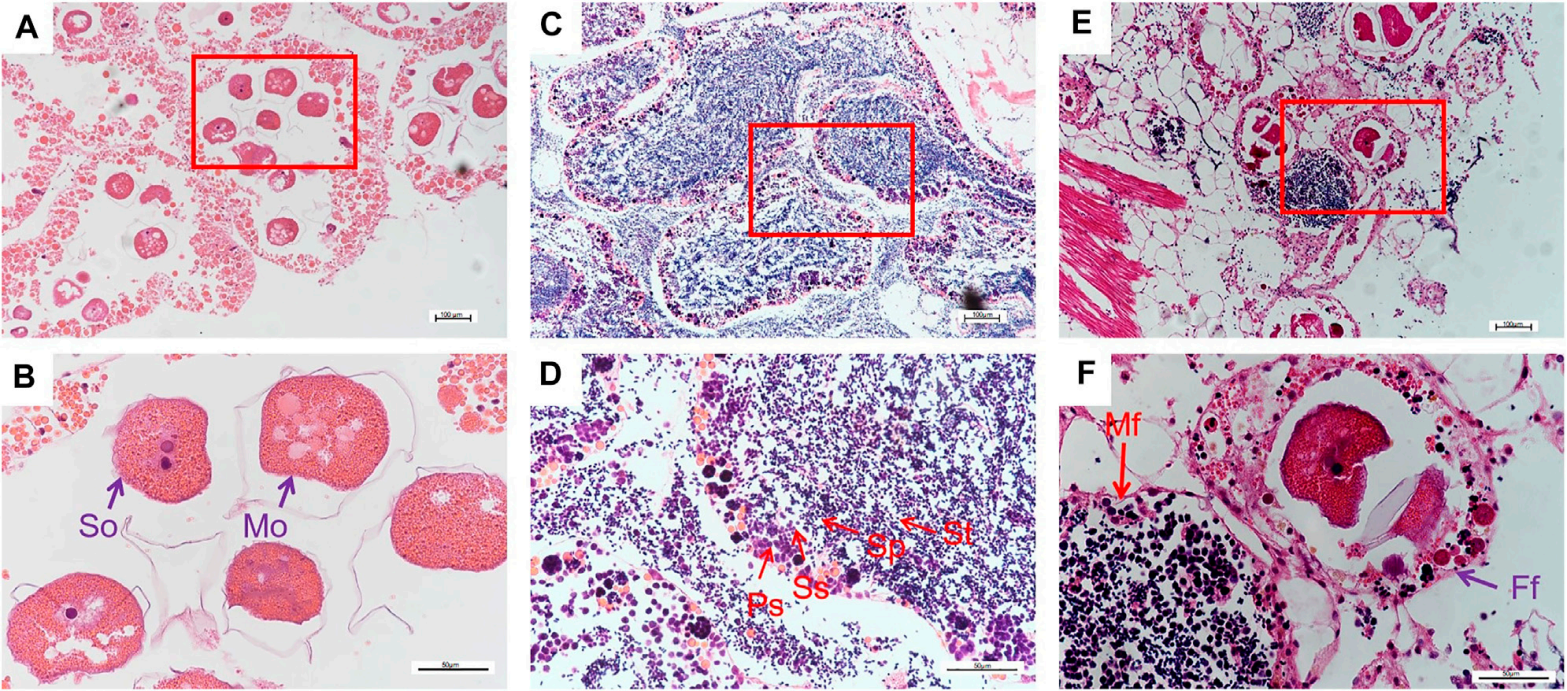
Histological characteristics of ovary **(A** and **B)**, testes **(C** and **D)** and hermaphrodite **(E** and **F)** from 30-month-old *H. schlegelii*.; So: Secondary oocyte; Mo: Mature oocyte; St: Spermatoblast; Sp: Sperm; Ss: Secondary spermatocyte; Ps: Primary Spermatocyte; M: Morula structure; Mf: Female follicle; Ff: Male follicle.

H&E staining for visualization in histological sections was used to examine the development of the gonads. The female follicles could be observed to contain secondary oocytes and mature oocytes. Histology identified each stage of sperm cells in follicles of male gonads, including primary spermatocytes, secondary spermatocytes, spermatoblast, and sperm. Meanwhile, the morula structure during sperm development also could be observed. Both female follicle and male follicle were clearly detected in the hermaphrodite gonads (Figure 1).

### Full-Length Transcripts From the Gonads of *H. schlegelii*

The full-length transcriptome of *H. schlegelii* was generated using the PacBio Sequel platform, transcriptomes were obtained for both testis and ovary. PacBio single-molecule real-time sequencing is circular sequencing, and the effective inserts read produced in the single-molecule detecting during the sequencing process are called subreads. The resulting total of 12, 478, 014 subreads based on 36.1G was generated by two SMRT cells from the PacBio library. The average length of the subreads was 2,889. Standard Iso-Seq self-correction, classification, and clustering protocols (see Materials and Methods) were applied to the sequencing results. The 899,605 circular consensus sequences (CCS) were produced through conditional screening (full passes of 2 and quality of 0.80). And there were 682,624 full-length non-chimeric reads after filtering for full-length read classifications, which contained both poly-A tail and two primers. Finally, the consensus sequences for the gonads of *H. schlegelii* included 359,889 transcripts (Table 1). Meanwhile, more than 4.8 × 10^8^ reads numbers and a Q30 of over 90% the high-quality clean short reads were generated from nine gonads of *H. schlegelii* using the Illumina sequencing platform (Supplementary Table S1).

**TABLE 1:** Description of Iso-Seq from the gonads of *H. schlegelii* by PacBio Sequel platform.

Species	Total gigabases (Gb)	Total subreads	Average subreads read length (bp)	Circular consensus sequence	Full-length non-chimeric reads	Average flnc read length	Consensus reads
*H. schlegelii*	36.04	12,478,014	2,889	899,605	682,624	3,308	359,889

**FIGURE 2:**
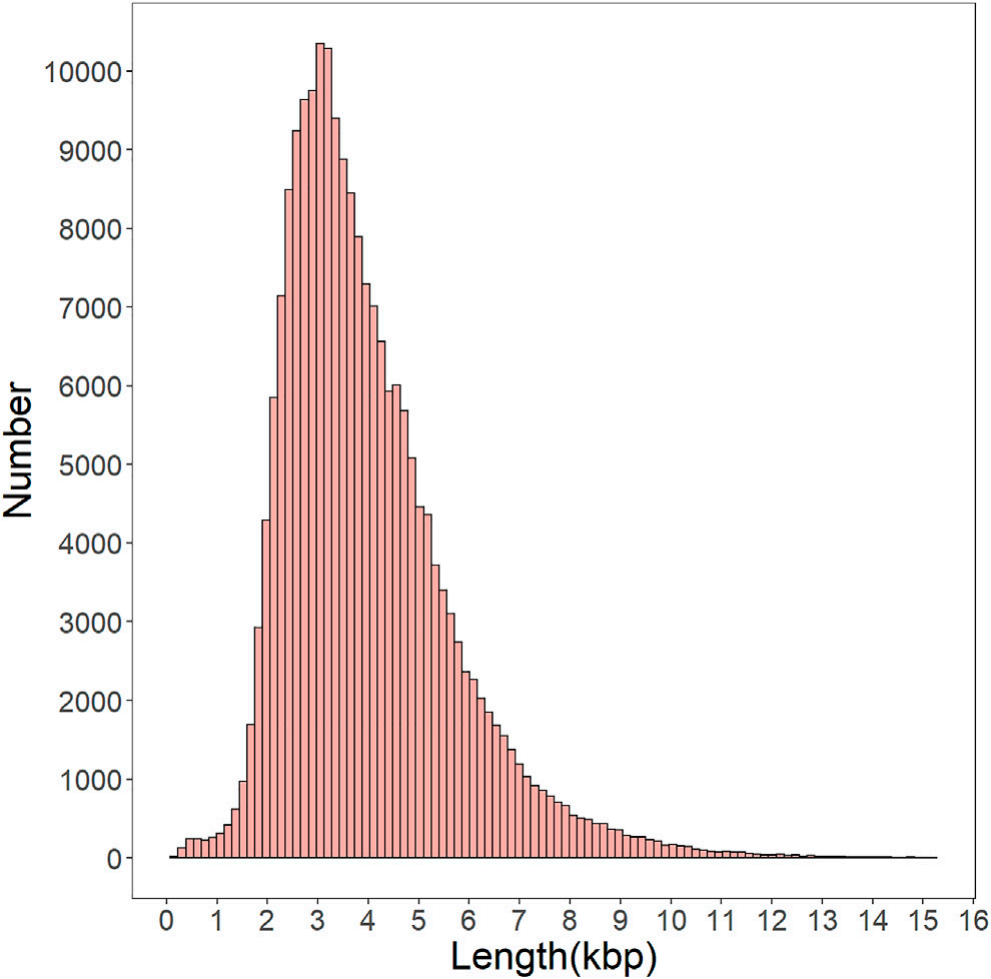
Distribution of nonredundant full-length transcript from the gonad transcriptome of *H. schlegelii*.

To build the full-length transcript with higher accuracy a lower error rate, all consensus sequences were corrected by short, clean reads as input data. After correcting the data, 359,889 transcripts, 3,779 N50, and 2,337 N90 were obtained (Supplementary Table S2). CD-HIT software removed redundant and similar sequences that resulted in 201,481 non-redundant transcripts with a mean length of 3,987 bp (Supplementary Table S3). About 99.2% of transcripts were longer than 1 kbp, and the main length distribution range from 2.5 to 5 kpb (Figure 2). These results show that the PacBio Iso-Seq is an efficient strategy for providing high-quality and full-length transcripts with Illumina short reads to correct.

### Gene Annotation of Full-Length Transcript From *H. schlegelii’s* Gonads

**FIGURE 3:**
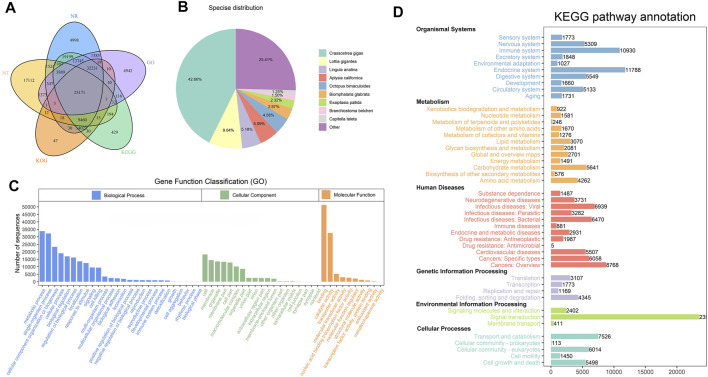
Annotation of transcripts from *H. schlegelii’*s gonads. **(A)** Gene function annotation in 5 databases (Nr, Nt, KOG, KEGG, GO). **(B)** Homologous species distribution of gonad annotated in the NR database. **(C)** Annotation of the GO function of the gonadal transcript. **(D)** Annotation of the KEGG function of the gonadal transcript.

For acquiring a comprehensive annotation of the *H. schlegelii* transcriptome, the 201,481 full-length transcripts were subjected to annotation analysis by aligning to public databases: NT NR, Swiss-Prot, KEGG, Pfam and GO. The Venn diagram shows that 78.31% (157,781) of the transcripts were successfully annotated in the least one database (Figure 3A). Optimal sequences alignments of sequence homology by NR database, 42.66% sequences had significant matches with *Crassostrea gigas;* 9.04% sequences were matched against *Lottia gigantea*, followed by *Lingula anatina* (5.18%), *Aplysia california* (5.09%), *Octopus bimaculoide* (4.58%). The remaining transcripts were homologous to those of other species (Figure 3B).

All the transcript annotated could be categorized into 55 GO terms (Figure 3C). Within the biological process category, the main GO terms were grouped in metabolic process, single-organism process and cellular component organization or biogenesis. Organelle was the most common annotation in cellular component category. Membrane and cell were the next most abundant GO terms. In the molecular function, the main proportion of GO terms referred to the binding and catalytic activity. Several putative hermaphroditism related to GO terms, including developmental process (995), reproduction (1092), and reproduction process (992), were significantly enriched.

A total of 61.65% transcripts from the gonad transcriptome of *H. schlegelii* were annotated with 46 subpathways from five pathways in the KEGG database (Figure 3D). The result indicated that signal transduction, endocrine system, and immune system were the top three pathways in abundance. Notably, some of these pathways may be involved in hermaphrodite, for example, oxytocin signaling pathway (1156), estrogen signaling pathway (965), and oocyte meiosis 734) (Supplementary Table S4).

### Search for Genes Involved in Hermaphrodite of *H. schlegelii*

A total of 87 candidate genes (1095) reportedly involved the gonad development, sex differences, and sex determination (Zhang et al., 2014; Yatsu et al., 2016; Li et al., 2020; Zhang et al., 2020; Broqurad et al., 2021) were present in the full-length gonad transcriptome of 30-month-old *H. schlegelii* (Supplementary Table S5). Four major sex determination-related genes in mollusks, *dmrt-like*, *sox9*, *wnt4*, and *fem1* were present in the full-length gonad transcriptome. Eight sex-related receptor genes were present, including the four-hormone receptor *esr1*, *gnrhr*, *lshr*, and *fshr*, and other four are *pgfra*, *spr*, *vgr*, and *zp3r*. Nine spermatogenesis-related genes were significantly matched including *atrx*, *boll*, *daz3*, *dnm3a*, *gtsf1*, *mycbp*, *piwl1*, *sycp1* and *scmh1* from the full-length gonad transcriptome. Meanwhile, *marf*, *vtg*, *hrom1*, *mta70* were associated with oogenesis, and the latter two were also related to spermatogenesis. Ten genes related to sex hormones synthesis and metabolism were identified. Four hydroxysteroid dehydrogenases *hsd17b2(17β-hsd2)*, *hsd3b1*, *hsd3b2*, and *hsd3b4*. The following six genes belonged to the cyp450 family: *cpy10*, *cyp17a1*, *cyp1a1*, *cyp1a2*, *cpy3a4*, *and cyp3a5*. Eight gonad development-related genes were also identified from the full-length gonad transcriptome, including the female gonad development genes cot*2*, *fst*, *vtg*, *vtg1*, *vtg6*, and *wnt4*, and genes involved in male gonad development, including *gata4* and *sox9*. Moreover, eight *Sapta* family genes and seven *Spag* family genes also were concerned. The remaining putatively hermaphrodite-related genes also appear in the Supplementary Table S5.

### Analysis of Candidate Long Non-coding RNAs, SSR and Transcription Factors

**FIGURE 4:**
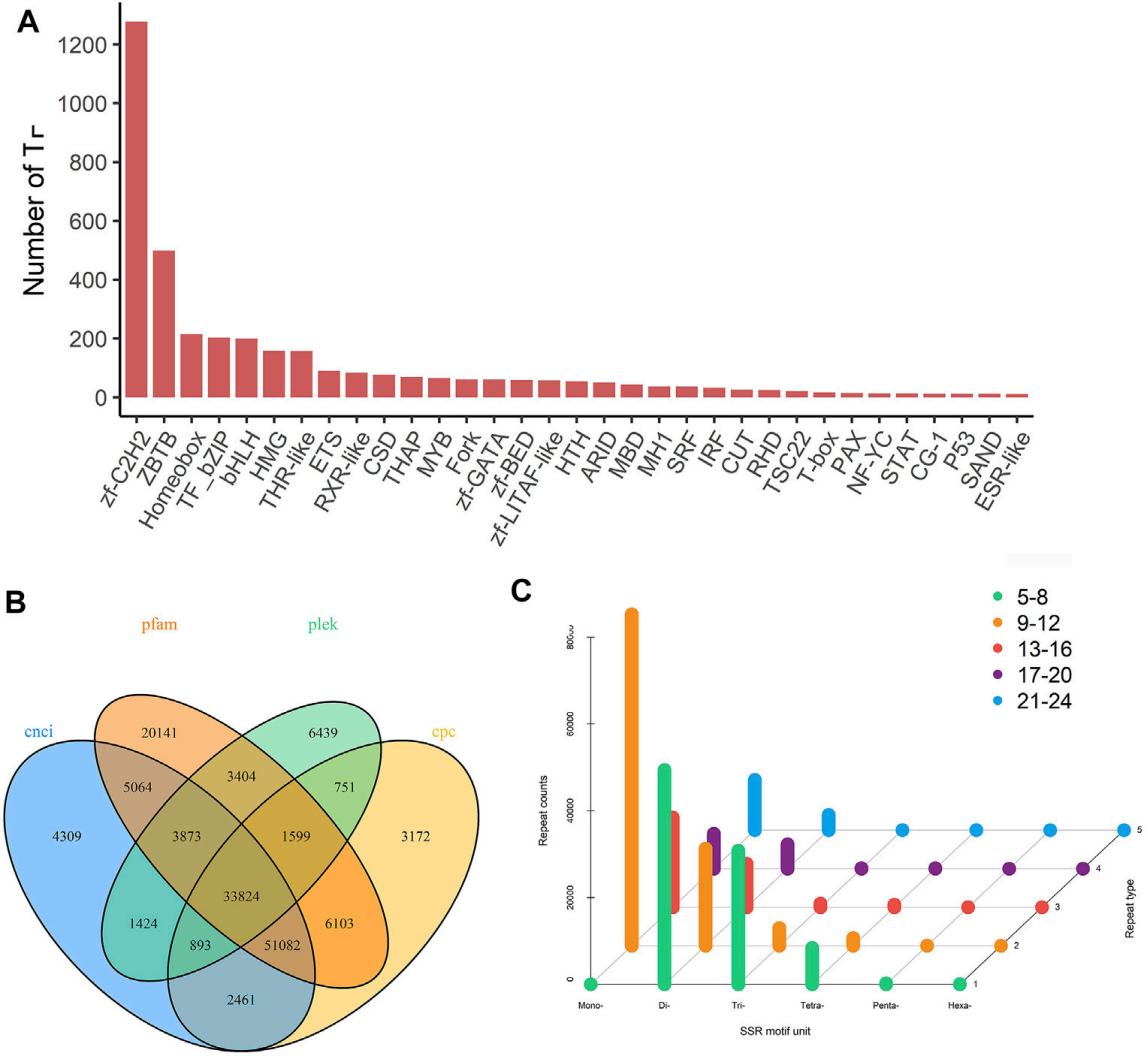
Prediction of transcription factors, long-non-coding RNAs and SSR of gonadal transcripts. **(A)** Transcription factor statistic. **(B)** Venn diagram of the number of lncRNAs predicted by CNCI, CPC, PLEK, and pfam protein structure domain analysis. **(C)** Prediction of SSR by MISA.

Transcription factors (TFs) play an important role in activating and inhibiting gene transcription, and they are also a current research focus. The animalTFDB2.0 database was used to identify the TFs of *H. schlegelii*, and the analysis predicted 61 TFs for gonads. The top 30 abundant terms are shown in (Figure 4A), of which the two most abundant were Zf-C2H2 and ZBTB. We used the unannotated transcripts to predict lncRNAs. The Venn diagram shows four methods for predicting the number of lncRNAs. Through CNCI, CPC, Pfam, and PLEK, 33,824 lncRNAs with high accuracy and reliability were identified (Figure 4B), these data aid in understanding how genes are expressed and regulated. More than 500 bp full-length transcripts were screened for SSR analysis. A total of 254,888 SSR sequences of six types (1–6 nucleotide repeats) were identified, and the distribution is shown in **(** Figure 4C). Single nucleotide repeats (9-12) and dinucleotide repeats (5-8) were the most common, with counts of 76,387 and 49,402, respectively.

### Differential Expression Genes (DEG) Analysis Involves in three Gonad’s Condition

**FIGURE 5:**
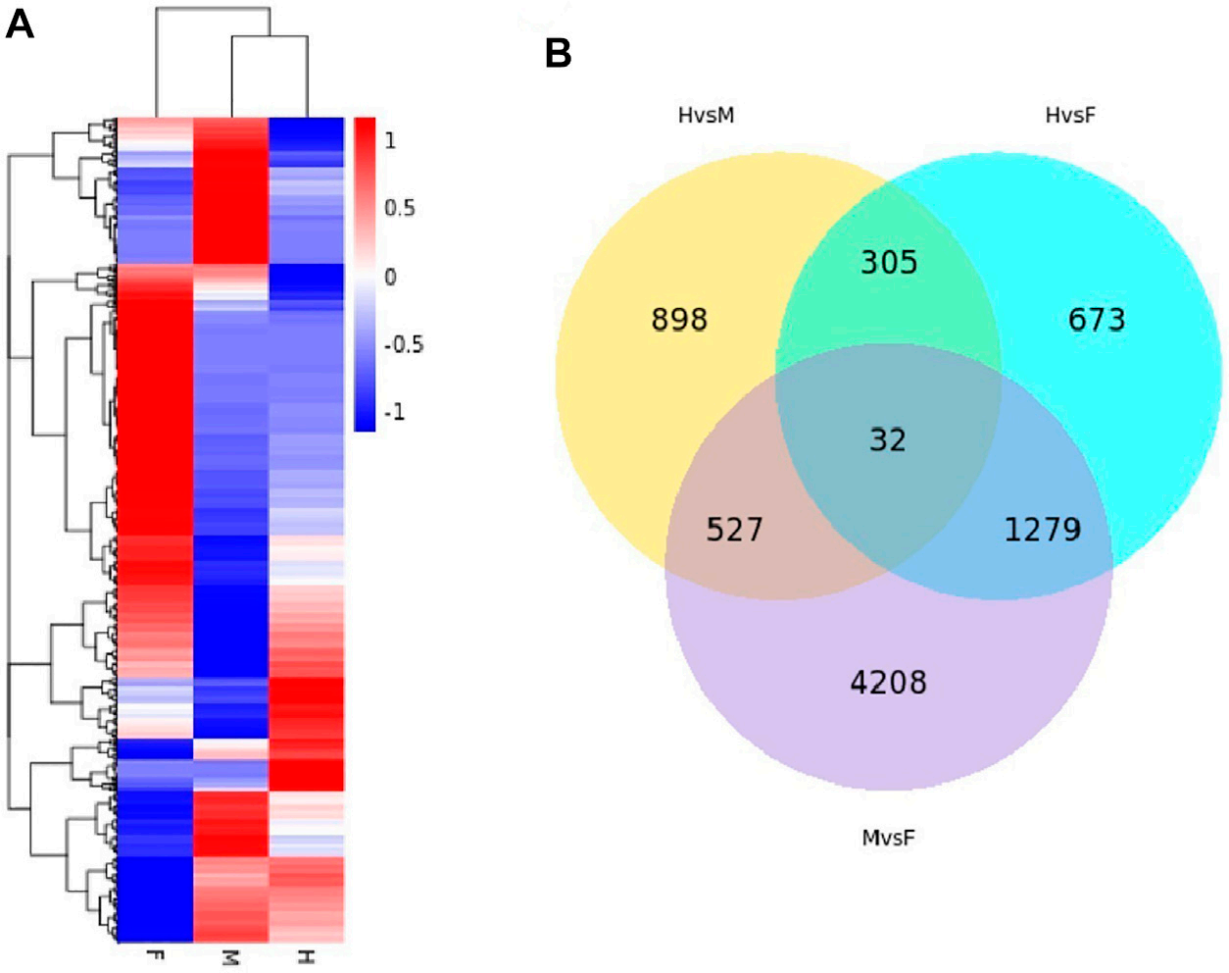
**(A)** The cluster heatmap (Log2(FPKM+ 1) values) indicates the expression patterns of deferential expression genes in the three type gonad of *H. schlegelii*. **(B)** Venn diagrams describing the numbers of unique and shared differentially expressed genes (DEGs).

Differential level expression of transcripts at three shapes of *H. schlegelii’s* gonad was tested using the program DEseq2, and the screening criteria were *Padj* < 0.05 and log_2_FoldChange >1. DEGs were detected between hermaphrodites and males (H vs. M), between hermaphrodites and females (H vs. F), and between males and females (M vs. F), with 2,289, 1,762, and 6,046 differentially expressed genes, respectively (Table 2). A total of 7,922 transcripts were expressed among all groups (Figure 5A). Hierarchical clustering was used to determine the distribution of DEGs among the three gonad forms (Figure 5B). Clustering showed that the male and hermaphrodite had similar expression levels, as they clustered into one branch.

**TABLE 2:** Result of differentially expressed gene.

	H Vs. F	H Vs. M	M Vs. F
UP	1588	892	2411
Down	701	870	3635
All	2289	1762	6046

**FIGURE 6:**
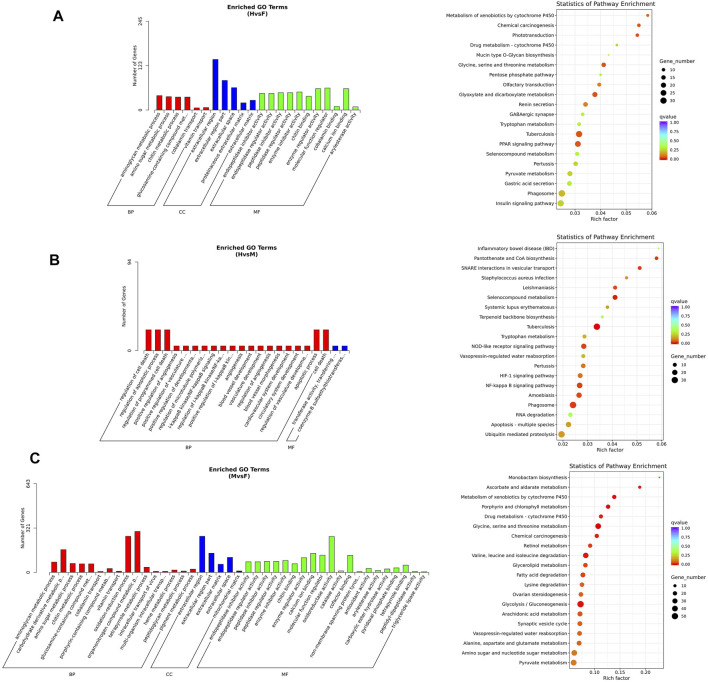
KEGG and GO enrichment analysis **(A)**GO analysis and KEGG analysis of DEGs under hermaphrodite and female. **(B)** GO analysis and KEGG analysis of DEGs under hermaphrodite and male. **(C)** GO analysis and KEGG analysis of DEGs under male and female.

For the hermaphrodite versus female group, the most enriched GO terms were “aminoglycan metabolic process” in the biological process category, “extracellular region” in the cellular component category, and “molecular function regulator” in the cellular component category. Most of the full-length transcripts were involved in the three top KEGG pathways, including “*tuberculosis*”, “glyoxylate and dicarboxylate metabolism” and “chemical carcinogenesis”. In the hermaphrodite versus male group, the most-enriched GO terms were “regulation of cell death”, “regulation of apoptotic process”, “regulation of programmed cell death” in the biological process category; “transferase activity, transferring alkylthio groups”, and “coenzyme-B sulfoethylthiotransferase activity” in the molecular function category. The KEGG enrichment analysis showed “*tuberculosis*”, “selenocompound metabolism”, and “phagosome” were the first three enriched pathways. For the male versus female group, the most-enriched GO terms were “organonitrogen compound metabolic process” in the biological process category, “extracellular region” in the cellular component category, “oxidoreductase activity” in the molecular function category. KEGG enrichment analysis concerned “glycine, serine and threonine metabolism”, “metabolism of xenobiotics by cytochrome P450”, and “porphyrin and chlorophyll metabolism” (Figure 6).

### Search for Differential Expression Genes and Hypothesized Pathway Related to Hermaphroditism of *H. schlegelii*

**FIGURE 7:**
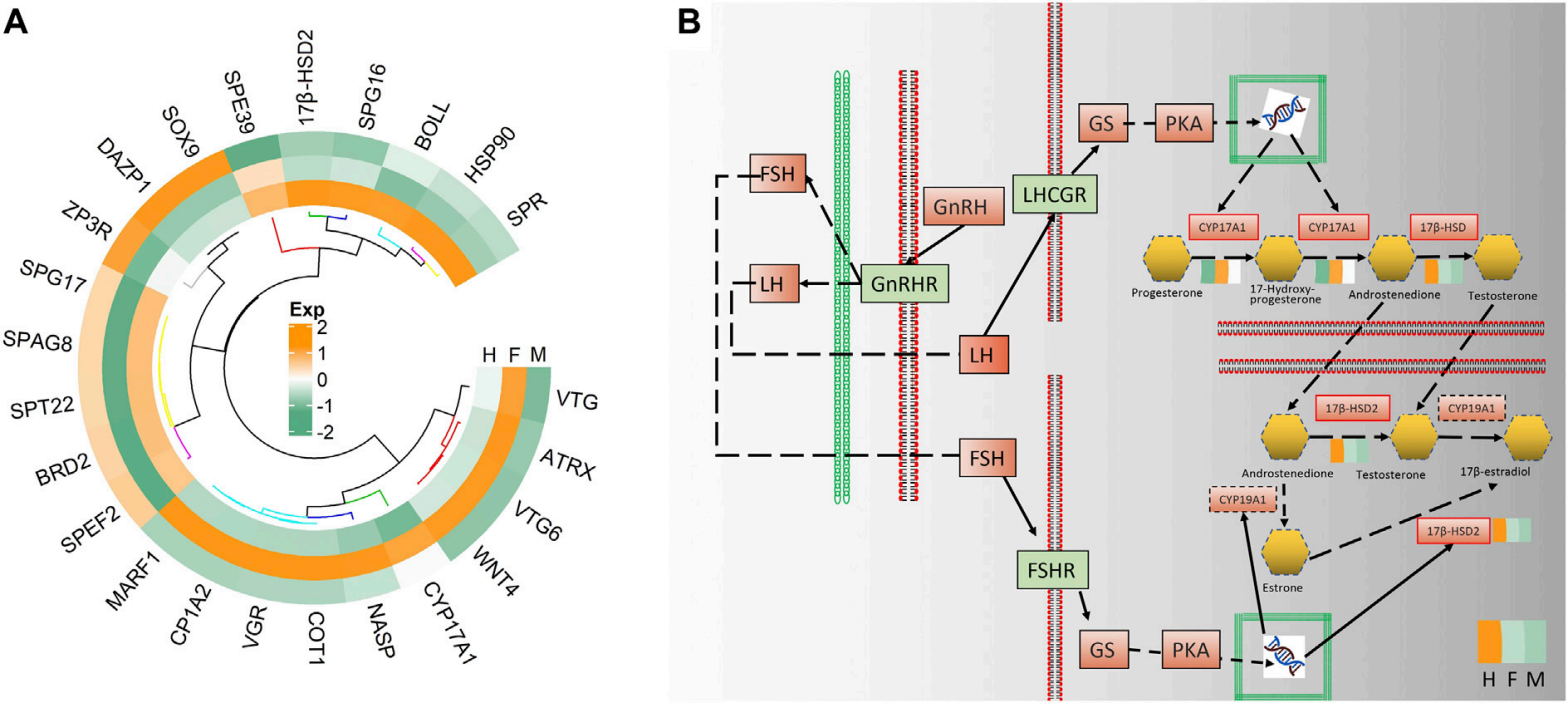
**(A)** The circle heatmap showed the expression level of the screened genes related to hermaphroditism. **(B)**The solid line and dotted line predicted interactions are displayed as gene-gene interactions; The solid line border indicates that the gene has been annotated, and the red solid line border indicates that the gene is a differentially expressed gene. The color blocks around the differentially expressed gene are the expression levels of the gene in the three types of gonads.

In further analysis 24 genes of the 87 early hermaphroditism-related genes were screened to have significant expression with three comparisons. Using FPKM as the expression level of 24 genes involved in hermaphroditism-related, make a gene expression heat map after normalization (Figure 7A). In total, ten genes (*vtg*, *atrx*, *vtg6*, *wnt4*, *cpy17a1*, *nasp*, cot*1*, *vgr*, *cyp1a2*, and *marf1*) were shown to be upregulated in the ovaries, while three genes (*sox9*, *dazp1*, and *zp3r*) exhibited higher expression in the testes. It is noteworthy of our attention that there are five genes (*17β-hsd2*, *spg16*, *boll*, *hsp90*, and *spr*) that are highly expressed in the hermaphrodite.

The genes *17β-hsd2*, *cpy1a2*, and *cpy17a1* were enriched in ovarian steroidogenesis, and each participates in sex hormone biosynthesis. Meanwhile, the full-length transcript is enriched to GnRH signaling pathway, oocyte meiosis, and steroid hormone biosynthesis. From these results, we may move forward in a single step to a hypothesized pathway for the formation of hermaphrodites in *H. schlegelii* (Figure 7B). *FSH* and *LH* are produced after being combined by *GnRH* and *GnRHR*. *LH* binds to *LHCGR* and activates the transcription of *cyp17a1*. *FSH* combinds *FSHR* to activate the transcription of *17β-hsd2* and *cyp19a1*. Under the action of *cyp17a1*, it catalyzes progesterone to produce androstenedione. However, there are two paths for androstenedione to generate 17β-estradiol. Androstenedione is catalyzed by *17β-hsd2* to testosterone, and then catalyzed by *cyp19a1* to 17β-estradiol. Another way is that androstenedione is catalyzed by *cyp19a1* to estrone, and then in 17β-estradiol was produced under the action of *17β-hsd2*. Both *17β-hsd2* and *cyp17a1* play important roles in the formation of sex hormones.

## Discussion

Second-generation sequencing technology has dramatically accelerated transcriptome research; even if the reads produced by the second-generation sequencing technology are short, they are generally very accurate. Because short reads will reduce transcript assembly accuracy, the development of third-generation sequencing technologies, such as those provided by the PacBio sequencing platform has significantly improved the *de novo* assembly of the transcriptome (Sharon et al., 2013), especially for species lacking a reference genome (Ferrarini et al., 2013). The transcriptome’s reconstruction and annotation play a crucial role in discovering genes and the in-depth study of the genomic characteristics of genomic species that have not been published (Hassan et al., 2012). Although the reads generated by third-generation sequencing have a high error rate, this strategy employed to correct sequencing errors can provide a high-quality and more complete integrated transcriptome in research by combining short and long-read sequencing technologies in a hybrid method (Hoang et al., 2017). This study combined RNA-seq and SMRT sequencing to generate a full-length transcriptome for *H. schlegelii* and comprehensively analyze the data. The results show that the transcripts sequenced using PacBio SMRT are much longer than sequenced using the Illumina platform. Compared with the previous research on the transcriptome of *H. schlegelii* using the Illumina platform (Shi et al., 2015), our study exhibits a more comprehensive transcriptome data set with the following highlights. First of all, the full-length transcripts we provided in this study can be used as a reference for developing the gene annotation of the *H. schlegelii*. Secondly, 96,867 and 111,520 accurate and high-quality transcripts were generated, providing valuable data for gene structure exploration and gene function research without using PCR. Finally, more SSRs, lncRNAs, and TFs can be used to evaluate the transcriptome of the gonads of *H. schlegelii*.

Sex development is a complex process, including gender determination, gender differentiation, and gender maintenance, until the final production of sperm or egg cells for reproductive system transmission (Zhang et al., 2016). Many genes work together to cause the gonads to differentiate into testes or ovaries, and subsequently cause the development of a male, female, or hermaphrodite phenotype (Li et al., 2020; Broquard et al., 2021; Yao et al., 2021). *H. schlegelii* has been reported to be a hermaphroditic species (Wu et al., 2017), its gonads are clearly visible at 12 month-old so that male and female can be divided; the gonadal tissue of *H. schlegelii* gradually grows steadily at 12–24 months of age, and the connective tissue is relatively abundant; Hermaphroditism was found at 28-34-month-old. In recent years, bivalves such as *Crassostrea gigas* (Broquard et al., 2021), *Tridacna squamosa* (Li et al., 2020), *Sinonovacula constricta* (Yao et al., 2021), *Nodipecten subnodosus* (Galindo-Torres et al., 2018), *Mytilus coruscus* (Kim et al., 2021), *Argopecten irradians* (Zhang et al., 2021) were reported to have the phenomenon of hermaphrodite. But the molecular mechanism of hermaphroditism in bivalve has rarely been studied, especially in freshwater mussel.

From the full-length transcriptome, 86 genes related to sex determination, gonadal development, and possibly related to hermaphroditism were found. A further 24 genes were detected and speculated to be related to gonadal development, including *sox9*, *wnt4*, *vtg, cyp17a1* and *17β-hsd2.*

We discovered the *sox9* was male-biased and thus may be a potential sex-determining gene in *H. schlegelii*. The HMG domain-containing transcription factor *sox9* plays an important role not only in testis-determining pathways of mammals and birds but also in male gonad differentiation and maintenance (Da silva et al., 1996). In *Hyriopsis cumingii* (Wang et al., 2021), *Pinctada margaritifera* (Teaniniuraitemoana et al., 2014) and *Sinonovacula constricta* (Yao et al., 2021), *sox9* also has been reported as a possible sex determination or sex differentiation gene. The *Cgsoxh*, homolog of the *sox9* in *Crassostrea giga*, was considered to be a regulator of *Cgdsx* and *Cgfolx2*, directly or indirectly affecting their interaction (Zhang et al., 2014). Previous studies have demonstrated that *wnt4* is closely related to the sex determination and differentiation of various animals (Bernard and Harley, 2007). *Wnt4* is a sex-determining gene and plays a vital role in sex reversal of mammals (Jordan et al., 2001). *Wnt4* is expressed in the gonads before sex determination in mice and continues be expressed in the ovaries after sex differentiation, but the testis expression is significantly reduced (Barrionuevo et al., 2006). In this study, we detected *wnt4* gene expression in the ovarian transcriptome of *H. schlegelii* but not in the testis, suggesting that *wnt4* may be related to female gonad determination and sex maintenance. Yolk formation, where the nutrients for embryogenesis are stored in the oocyte, is one of the key steps in oogenesis. The vitellogenin (*vtg*) transcript of *Patinopecten yessoensis* was reported to be abundantly expressed in female gonads by transcriptome sequencing techniques (Hou et al., 2011; Zhou et al., 2019). *Vtg* mRNA expression was restricted to the ovary of *Crassostrea angulata*, where it was thought to be synthesized and controlled by 17β-estradiol (Ni et al., 2014). In *Chlamys nobilis* transcriptomic studies, it has also been shown that the expression is significantly higher in females than in males (Sigang et al., 2021). A large number of *vtg* transcripts was found in gonadal trancriptome, and the expression of *vtg* was much higher in female than in male. We inferred that *vtg* acts a pivotal part in female development.

*Cyp17a1* and *17β-hsd2* play crucial roles in the putative pathways involved in hermaphroditism. The *cyp17a1* gene is primarily related to endocrine function and steroid hormone metabolism and is involved in the biosynthesis of sex hormones (Ma et al., 2016). It is a microsomal enzyme with both 17α-hydroxylase and 17,20-lyase activities (DeVore and Scott, 2012). The enzyme is involved in the production of steroid hormones such as estrogen, androgen, and cortisol. *Cyp17a1* can catalyze progesterone to 17-hydroxy-progesterone, which further generates androstenedione (Petrunak et al., 2014). *Cyp17a1*-like plays a critical role in Cd detoxification, and knockdown of *cyp17a1*-like led to a significant increase in mortality and Cd content in *Crassostrea gigas* (Tian et al., 2021). *Cyp17a1* could be an important steroidogenesis enzyme to coordinating testosterone in probable GnRH and steroidogenesis pathways of *Patinopecten yessoensis* (Zhang et al., 2020). The present results indicate that the expression of *cyp17a1* was the highest in females. It is speculated that *cyp17a1* is involved in sex steroid hormone production, and *cyp17a1* catalyzes more progesterone to prepare for the production of estradiol that in turn generates more estrogen and then promotes ovarian development.

The enzyme *17β-hsd2* is the major oxidase in the *17β-hsd* family that catalyzes the interconversion of testosterone and androstenedione as well as estradiol and estrone (Wu et al., 1993). In addition, *17β-hsd2* is also involved in the inactivation of androgen and estrogen (Moeller and Adamski, 2006). We found the *17β-hsd2* was highest in hermahprodites, and *17β-hsd2* apparently plays a crucial function in our hypothetical pathway. Although *cyp19a1* was not found in the gonadal transcriptome of *H. schlegelii*, *17β-hsd2* is still an essential enzyme for the conversion of androstenedione to estradiol. The increase of *17β-hsd2* may generate more estradiol to facilitate the occurrence of female follicles. This study verified the conjecture that the hermaphrodites of *H. schlegelii* have developed from males.

## Conclusion

The present study provides the new genetic resources of comprehensive full-length gonad transcriptome of the *H. schlegelii*, an economically important cultured freshwater pearl mussel species. The results showed that a total of 201,481 high-quality nonredundant full-length transcripts were generated from 36.04 G subread bases with an average length of 3,987 bp. The TF, SSR and lncRNAs of *H. schlegelii* were predicted, representing a major advance in freshwater shellfish genetics. In addition, the number of 86 genes were identified as related to gonadal development, of which 24 genes were differentially expressed in the three comparison groups, indicating important functions in the gonadal development of *H. schlegelii* included *sox9*, *wnt4*, *vtg*, *cyp17a1* and *17β-hsd2* for sex differentiation and gonadal development. Meanwhile, we also speculated on possible pathways for the formation of hermaphroditism. On balance, our research provides the first full-length gonad transcriptome of bivalves, and the results provide important candidate genes and a theoretical basis for further exploring the gonadal development and hermaphroditism in related bivalves.

## Data Availability

The datasets presented in this study can be found in online repositories. The names of the repository/repositories and accession number(s) can be found below: SRA; PRJNA806470, PRJNA806375.
